# A large-scale targeted proteomics of serum and tissue shows the utility of classifying high grade and low grade meningioma tumors

**DOI:** 10.1186/s12014-023-09426-9

**Published:** 2023-09-29

**Authors:** Ankit Halder, Deeptarup Biswas, Aparna Chauhan, Adrita Saha, Shreeman Auromahima, Deeksha Yadav, Mehar Un Nissa, Gayatri Iyer, Shashwati Parihari, Gautam Sharma, Sridhar Epari, Prakash Shetty, Aliasgar Moiyadi, Graham Roy Ball, Sanjeeva Srivastava

**Affiliations:** 1 Department of Biosciences and Bioengineering, Indian Institute of Technology Bombay, Powai, Mumbai, 400076 India; 2 Motilal Nehru National Institute of Technology, Allahabad, 211004 UP India; 3 Department of Bioscience & Bioengineering, Indian Institute of Technology Guwahati, Guwahati, 781039 Assam India; 4 CSIR-Institute of Genomics and Integrative Biology, Sukhdev Vihar, New Delhi, 110025 India; 5 Institute for Systems Biology, 401 Terry Ave N, Seattle, WA 98109 USA; 6 Koita Centre for Digital Health, Indian Institute of Technology Bombay, Powai, Mumbai, 400076 India; 7 Department of Pathology, Tata Memorial Centre, Mumbai, India; 8 Department of Neurosurgery, Tata Memorial Centre, Mumbai, India; 9 Medical Technology Research Centre, Anglia Ruskin University, Cambridge Campus, East Rd, Cambridge, CB1 1PT UK; 10 Department of Bioengineering and Therapeutic Sciences, University of California, San Francisco, 185 Berry St., Suite 290, San Francisco, CA 94107 USA

**Keywords:** Meningioma, High grade tumors, Low grade tumors, Glioblastoma/Glioma, Targeted Proteomics, Selected/Multiple reaction monitoring (SRM/MRM)

## Abstract

**Background:**

Meningiomas are the most prevalent primary brain tumors. Due to their increasing burden on healthcare, meningiomas have become a pivot of translational research globally. Despite many studies in the field of discovery proteomics, the identification of grade-specific markers for meningioma is still a paradox and requires thorough investigation. The potential of the reported markers in different studies needs further verification in large and independent sample cohorts to identify the best set of markers with a better clinical perspective.

**Methods:**

A total of 53 fresh frozen tumor tissue and 51 serum samples were acquired from meningioma patients respectively along with healthy controls, to validate the prospect of reported differentially expressed proteins and claimed markers of Meningioma mined from numerous manuscripts and knowledgebases. A small subset of Glioma/Glioblastoma samples were also included to investigate inter-tumor segregation. Furthermore, a simple Machine Learning (ML) based analysis was performed to evaluate the classification accuracy of the list of proteins.

**Results:**

A list of 15 proteins from tissue and 12 proteins from serum were found to be the best segregator using a feature selection-based machine learning strategy with an accuracy of around 80% in predicting low grade (WHO grade I) and high grade (WHO grade II and WHO grade III) meningiomas. In addition, the discriminant analysis could also unveil the complexity of meningioma grading from a segregation pattern, which leads to the understanding of transition phases between the grades.

**Conclusions:**

The identified list of validated markers could play an instrumental role in the classification of meningioma as well as provide novel clinical perspectives in regard to prognosis and therapeutic targets.

**Supplementary Information:**

The online version contains supplementary material available at 10.1186/s12014-023-09426-9.

## Introduction

The discovery of cancer biomarkers has played a massive role in improving early screening and diagnosis, risk stratification, determining response to certain medications, monitoring disease progression and predicting prognosis. Over the past decade, advancements in mass spectrometry have significantly boosted the field of proteomics. As a result of these advances, proteomics has now forayed into clinical practice in comprehending the biology of diseases like cancers and infectious diseases [1]. It has accelerated the discovery of biomarkers, improvement of therapy modalities and the identification of new drugs. The advent of various consortia like CPTAC (Clinical Proteomics Tumor Analysis Consortium) and HUPO (Human Proteome Organization) has further unleashed the power of proteomics in clinical applications [2–4]. At the data analysis front, the recent advancements in several stand-alone tools and databases have facilitated the development of integrated omics pipelines and meta-analysis workflow that expedites the understanding the disease pathophysiology, identification of newer biomarkers, and predicting novel therapeutic modalities [5, 6]. A perfect cancer biomarker is defined as one that is highly specific, selective, easily detectable at an early stage of the disease, and measurable at a low cost, however, identifying a biomarker with all these desired features is a highly arduous task [7, 8]. The early diagnosis and treatment of brain-related tumors and other cancers continues to be challenging even today. Despite many advancements, there have been multiple challenges in clinical translation of many potential biomarkers.

Meningiomas are one of the most frequently occurring intracranial tumors accounting for around 37% of all brain tumors [9]. Meningiomas arise from meningeal layers and according to the recent classification by World Health Organization (WHO) are divided into three grades (I, II, and III) [10] based on their histopathological features. Though most meningiomas remain benign [9], about 1–3% turn malignant with good survival rate, however, surgical resection of tumors present near crucial brain regions pose a greater challenge to the operating surgeon [11]. Radio-diagnostic techniques like MRI and CT scans are majorly employed for the prefatory diagnosis of meningiomas [12] aiding in stratifying tumors into two types based on their location, viz. skull base and convexity. However, there still remains a need to identify biomarkers that can help in early diagnosis and predicting prognosis.

The recent advancements in Omics-based technologies have facilitated the identification of various biomarkers and demonstrated their role in understanding tumorigenesis and progression. On the genomics front, loss of heterozygosity (LOH) in chromosome 22q and causative variants in a tumor suppressor gene, neurofibromatosis type 2 (NF2), have been associated with the formation of Meningiomas [13, 14]. Recent studies have also illustrated the mutations in TERT promoter regions in grade III meningiomas, along with alterations in SMARCE1 and BAP1 that have been reported widely in clear cell and the rhabdoid subset of meningiomas [15, 10]. Comprehensive methylome profiling led to dividing meningioma into six sub-classes and predicting survival outcomes [16]. Meanwhile, there are also parallel efforts undertaken at the proteomics front to comprehend the pathophysiology of meningioma and identify prognostic markers using high-throughput technologies. Studies have revealed the perturbations in PI3K/AKT pathways and different signalling cascades, along with identifications of a number of differentially regulated proteins in diseased states like EGFR, CKAP4, NEK9, SF2/ASF (splicing factor) and HK2 [12, 17]. Several groups have attempted to identify grade-specific protein markers in the serum but need more comprehensive quantification and validation in larger cohorts and across grades. Proteins like Apolipoprotein E and A-I, hemopexin, alpha-2-macroglobulin, apolipoprotein B, and antithrombin-III have been reported as predictive markers [18]. Similarly, immunoassays have revealed a set of proteins like amphiregulin, CCL24, CD69, Prolactin, and caspase-3 to be upregulated in Meningioma [19]. Autoantibody screening has also revealed proteins like IGHG4, CRYM, EFCAB2, STAT6, CCNB1, etc., to be differentially regulated across grades of Meningioma [20].

Immunoassays are widely applied in conventional clinical diagnostics settings for their sensitivity and robustness. While immunoassays like ELISA are considered as the gold standard for routine clinical settings [21]; the associated challenges like cross-reactivity, limited throughput, low sensitivity, time and labour intensiveness, have compelled clinicians to seek and researchers to shift their focus towards developing MS-based clinical assays. Because of the high throughput nature and the rapid development of MS technologies are fostering Point Of Care (POC) devices in clinical settings to directly analyze biological materials like blood, tissue, sweat, urine, saliva, etc. [22, 23]. Similarly, the targeted proteomics approaches like the Multiple (or selected) reaction monitoring-mass spectrometry (MRM/SRM) have been employed to validate proteomic biomarkers. Numerous studies have implemented MRM/SRM methods for relative and absolute quantification) of proteomic biomarkers in biofluids like plasma, urine [24, 25] as well as tissues [26]. The multiplexed SRM assays are extensively used and found to be highly reliable and reproducible for the sensitive and accurate quantification of proteins [27]. A significant achievement is standardizing the analysis of vitamin D levels in blood serum [28].

In this study, we have validated a list of potential markers reported in numerous manuscripts, popular knowledgebases, and data repositories using 53 fresh frozen tissue and 51 serum samples of Indian origin. The study optimized and selected unique peptides for each protein reported and verified using an MRM-based targeted proteomics approach. In addition to this, data analysis strategies like discriminant analysis, statistical analysis, feature selection and ML approaches were implemented to identify a list of 15 markers from tissue and 12 markers from serum, with ~ 80% accuracy in predicting high grade and low grade meningioma despite huge heterogeneity, sub-types and transition phases between the grades. This is one of the first studies which has attempted to verify and validate reported markers in such a large number of fresh frozen tissue and serum of Meningioma, which could be referred to as proof of concept to move forward in clinical diagnosis of meningioma.

## Materials and methods

### Ethics approval and informed consent

The study has been approved by the Institutional Ethics Committee of the Advanced Centre for Treatment Research and Education in Cancer (ACTREC), Tata Memorial Hospital (TMH), Mumbai, India, and IIT Bombay (ACTREC-TMC IEC No.149). The participants provided their due consent for participation in the study.

### Sample collection

All the experiments were performed in accordance with the Institute’s Biosafety Guidelines. Surgically resected tumor tissues and serum (from peripheral blood collected during the surgery) were taken for proteomics analysis. Surgically resected tumor tissues were collected from 23 low grade Meningiomas (WHO grade I) and 18 high grade Meningiomas (grade II and III), compared with 8 control tissues and 4 Glioma/Glioblastoma tissue samples. Serum samples were collected from 17 low grade Meningioma (WHO grade I) and 14 high grade Meningioma (WHO grade II and III) samples, along with 6 healthy controls and 14 Glioblastoma samples. The relevant clinical information is provided in Table S1. In this study, the experimental pool samples were prepared by pooling all samples and subjected to protein extraction and data acquisition to monitor the instrumental variation as well as to analyse the stability of the peptides.

### Mining and preparation of protein list

Text-mining, literature survey, popular knowledgebases curation, and results of discovery proteomics studies were thoroughly checked to develop a list of protein markers linked to Meningioma pathogenesis. The proteins were further mapped with the biological pathways and diseases-gene association networking hubs to narrow the list. The knowledge bases used for the curation of markers are eDGAR [29], PubPular [30], DisGeNET [31], BIONDA [32], and Harmonizome [33]. In addition, ProteomeXchange [34], PRIDE [34], OmicsDi [35], and Pubmed [36] were also used to mine the proteomics studies related to Meningioma. The biological interpretation was made using DisNor [36], KEGG [37], and Meta-Scape databases [38].

### Preparation of tissue and serum samples

The samples from fresh frozen tumor tissues and undepleted serum samples were prepared for proteomic analysis, as previously reported [39, 26]. The protein from tissue specimens (~ 50 mg) was extracted with the lysis buffer composed of 8 M urea, Tris-HCl buffer (pH 8.0), and a Protease Inhibitor Cocktail (PIC) (Sigma Aldrich®) complex. 50 ug proteins from both serum and tissues were reduced with TCEP, followed by alkylation with iodoacetamide (IAA). The reduced and alkylated proteins were subjected to enzymatic digestion by trypsin (Pierce, Thermofisher Scientific). After overnight incubation for 16 h at 37 °C, the digests were concentrated by vacuum drying and reconstituted in 0.1% (v/v) formic acid (FA). The *in-house* C18 stage tips were used for desalting. The desalted peptides were further dried and reconstituted using 0.1% (v/v) FA. The Scopes method was used for quantifying the peptides by measuring O.D. values at 205 and 280 nm.

### Transition list preparation

Proteins and their peptides for MRM experiments were selected based on published data, submitted discovery experiments, and information available in SRM Atlas. The transition list was prepared in Skyline daily using the Uniprot accession IDs for the target protein taking the human UniProt database as background proteome. Peptide length was set to 8–20 amino acids [40]. The peptides were filtered out based on uniqueness for each protein checked from NextProt [41] and 0 missed cleavages. The transition list included y-ions from “first ion” to “last ion.” corresponding to + 2 and + 3 precursor ion charge. Method files were created for the unrefined transition list for the selected proteins. Initial optimization was performed using the sample pool to select the peptides and their transitions for each protein. The optimal peptides and their transition for each protein were chosen by monitoring the sample pool. The transition list of the peptides for both tissue and serum is available in Table S2 and S3 respectively.

### Data acquisition using LC/MS

The data was acquired using a TSQ Altis Mass Spectrometer (ThermoFisher Scientific) connected with an HPLC system (Dionex Ultimate 3000 -ThermoFisher Scientific). Peptides were separated using Hypersil Gold C18 column (1.9 μm, 100 × 2.1 mm, ThermoFisher Scientific). MRM runs were performed using a flow rate of 450 µL/min, a cycle time of 2 s, and a resolution of 0.7 m/z (Q1 and Q3) over an LC gradient of 10 min. The solvent system included 0.1% FA and 100% Acetonitrile (ACN). The data obtained was further analyzed using Skyline daily where peak selection and refinement were made based on considering the peak shape, dot product and retention time. For further statistical analysis, the values for peak area were exported, and peptide-wise comparison was made.

### Experimental design for tumor tissue and serum sample

**Fig. 1:**
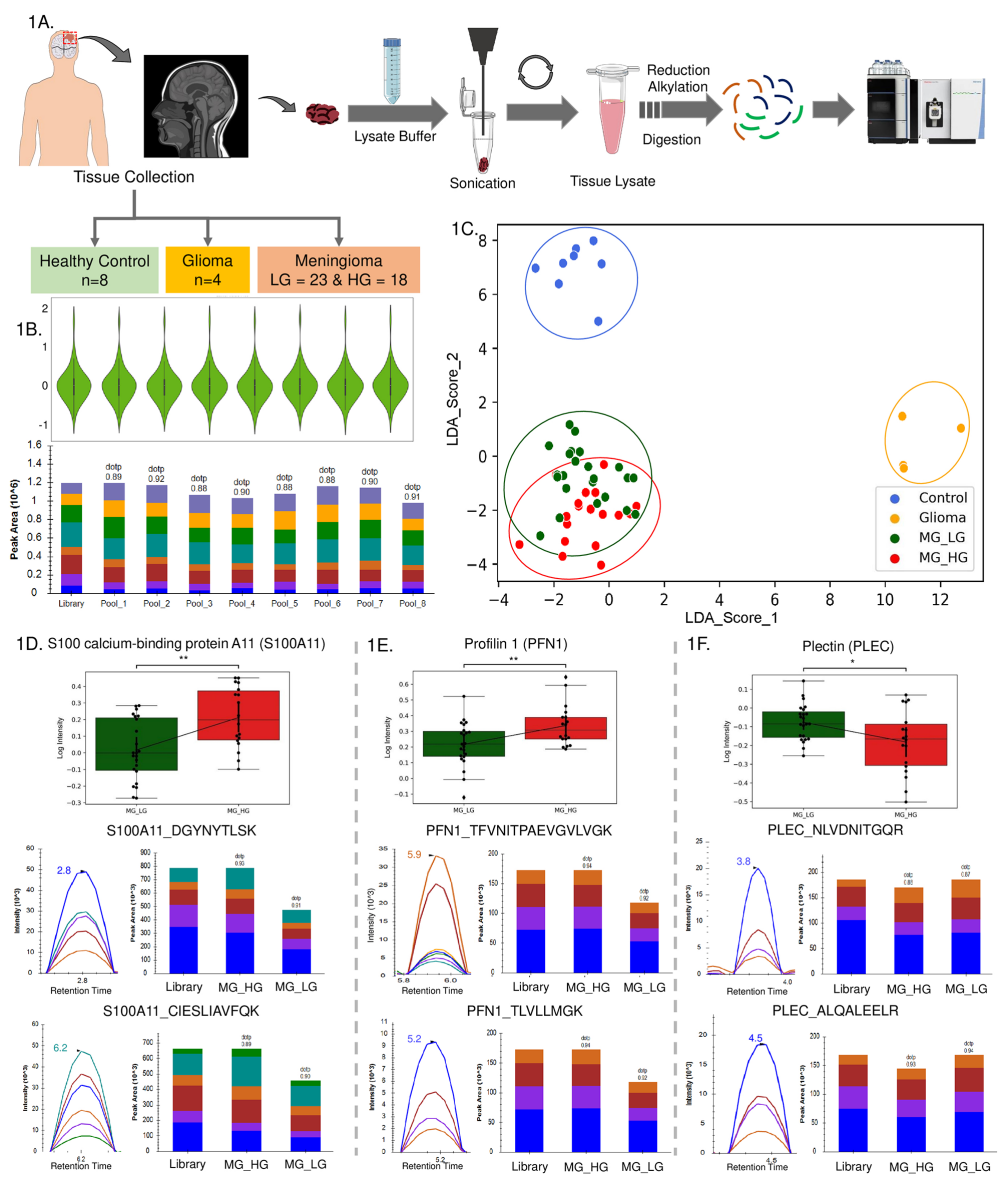
Validation of proteins using MRM assay to understand the segregation of Meningioma grades in the Tissue sample. (**1A**) Schematic outline of setting up of targeted proteomics of Tissue samples. (**1B**) QC Profile plot of all the peptides in the Pool samples to decipher the integrity and stability of the assay in intra and inter-day comparison, (**1C**) LDA score-based clustering depicting the segregation of the sample groups; (**1D-F**) represents the peaks and expression of S100A11, PFN1, and PLEC using profile plot between High-Grade Meningioma vs. Low-Grade tissue samples

**Fig. 2:**
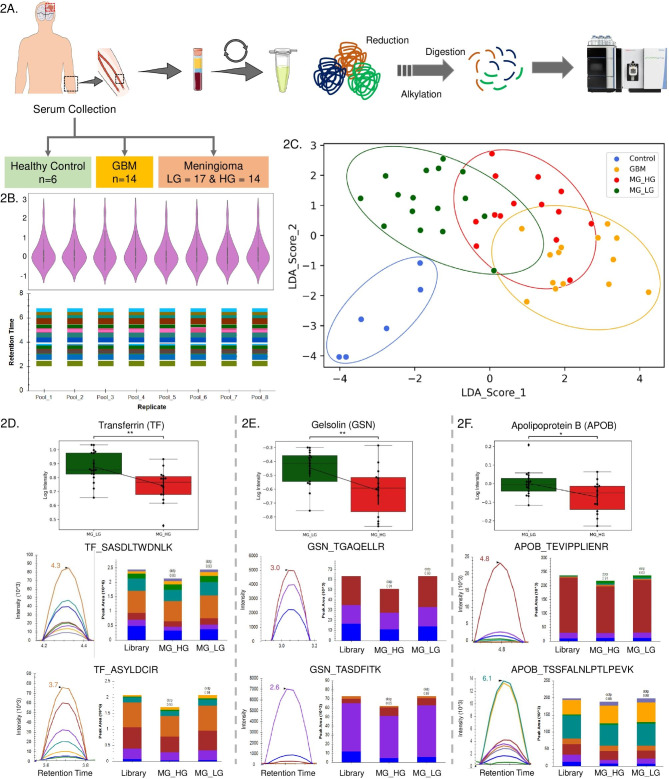
Validation of proteins using MRM assay to understand the segregation of Meningioma grades in the serum sample. (**2A**) Schematic outline of setting up of targeted proteomics of serum sample. (**2B**) QC Profile plot of all the peptides in the Pool samples to decipher the integrity and stability of the assay in intra and inter-day comparison, (**2C**) LDA score-based clustering depicting the segregation of the sample groups; (**2D-F**) represents the peaks and expression of TF, GSN, and APOB using profile plot between High-Grade Meningioma vs. Low-Grade serum samples

A total of 49 and 24 selected proteins with more than 2 unique peptides and at least 3 transitions for each peptide were optimized in multiple pools and analysed in 53 tissue and 51 serum samples respectively. The tissue samples included 8 controls, 4 Glioma, 23 low grade meningioma, and 18 high grade meningioma while serum samples included 6 controls, 14 GBM, 17 low grade meningioma, and 14 high grade meningioma (Table S1). The schematic outline of the experimental plan for the targeted proteomics-based validation of tissue and serum samples has been provided in Fig. 1 A and 2A respectively.

### Feature selection and machine learning (ML) based analysis

The analyzed files containing peak area values were exported from the Skyline daily into an Excel sheet for further ML and statistical analysis. The peptide correlation of pool samples was performed to check for the instrument variability (Fig. S1A and Fig. S2A). The peptides have been filtered based on the coefficient of variation (CV) values in QC-Pool samples. Statistical analysis and data visualization were carried out in Python and Microsoft Excel. The Linear Discriminant Analysis (LDA) plot was used to understand the cohorts. The Support Vector Machine (SVM) classifier was used to classify the low grade patients from high grade patients after comparing different classifier algorithms like k Nearest Neighbor (kNN), Random Forest, Naive Bayes, and Logistic Regression algorithm. K-fold cross-validation (*k* = 10) was employed in evaluating the models’ performance, in which the data was split into k randomly chosen subsets of about equal size. One subset was then used to validate the trained model using the remaining subsets. Finally, the average of all k subsets was taken to evaluate the final model performance score. The SVM linear model performance was further evaluated and visually represented by plotting the ROC-AUC curve and confusion matrix using Python-based tools.

## Results

In this study, we identified a panel of proteins from tissue and serum samples that can segregate meningioma patients into low grade patients and high grade with around 80% accuracy. This report, to our knowledge, has for the first time attempted to verify and validate the potential of identified meningioma markers using robust, targeted proteomics. This study thus lays the foundation to move a step forward towards the diagnosis as well as prediction of disease prognosis of meningioma using targeted proteomics approach.

### Quality control check and assessment of instrumental variation through pooled tumor samples

A pool sample consisting of all the tissue samples and all the serum samples were prepared and run in-between the samples to evaluate the instrumental variability (Figs. 1B and 2B). A correlation coefficient greater than 0.95 (Fig. S1A and B, Fig. S2A and B) depicts that there was no significant variability during data acquisition and the instrument performance was comparable throughout the experiment.

### Linear discriminant analysis segregates the meningioma samples cohort

The LDA of 53 tissue and 51 serum samples was performed with the list of 183 and 118 peptides from 49 and 24 proteins respectively to understand the segregation between different cohorts. From the linear discriminant analysis, it is quite evident that the expression level of the studied peptides and proteins in the cohort of meningioma low grade and high grade samples showed remarkably different characteristics in the control and glioma sample cohort. However, the segregation between low grade and high grade meningioma was unclear and found to overlap (Fig. 1C). About 28.8% of the features showed a maximum classification of high grade samples from low grade samples, keeping a mixed population in between (Fig. S1C). The classification plots showed some low grade and high grade samples in the extreme of the two cohorts. However, some samples appeared at the intersection of the two grades. These samples showed the heterogeneity in meningioma which could be a result of the tumors transitioning from one grade to the other.

### Alteration of tissue proteomics markers with respect to meningioma tumor progression and pathogenesis

The partial segregation of the meningioma grades using the LDA plots shows the commonalities and correlation between the samples despite being two different clinical grades. The classical strategy of identification of differential expression with significant t-test analysis identifies promising grade-specific tissue markers due to the biological variation and heterogeneity of the samples. Proteins like Profilin 1 (PFN1), H4 Clustered Histone 1 (H4C1), Annexin A1 (ANXA1), S100 calcium-binding protein A11 (S100A11) and Lactate Dehydrogenase (LDH1) were found to be upregulated in high grade Meningioma in comparison to low grade. However, Plectin (PLEC) and Mucins (MUC4, MUC5A, MUC1) were found to be downregulated in high grade Meningioma. The sample-wise expression of these proteins has been shown as a heatmap (Fig. S1D). The differential expression of proteins, like S100A11 (DGYNYTLSK and CIESLIAVFQK), PFN1 (TFVNITPAEVGVLVGK and TLVLLMGK), and PLEC (NLVDNITGQR and ALQALEELR) with their peptides can be found in Fig. 1D, E, and F. The expression level of peptides and proteins are provided in Table S4.

### Segregation profile of different patient cohorts deciphers the potential of serum protein markers

A list of 24 proteins after peak refinement having more than 2 peptides and atleast 3 transitions for each peptide was taken for LDA to understand the segregation of healthy control, GBM, low grade, and high grade meningioma samples. The LDA plots showed clear segregation between healthy control samples from the meningioma and GBM sample cohorts. The high grade meningioma sample cohort overlapped with the low grade meningioma and GBM cohort (Fig. 2C). Around 23.5% of the peptides showed segregation between the meningioma grades (Fig. S2C).

### Differential expression analysis to identify altered serum markers in Meningioma

Normalised peak areas were used to calculate the fold change for identifying the differentially expressed proteins. Proteins like Transferrin (TF), Apolipoprotein B (APOB), Cytochrome c oxidase subunit III (CO3), and Albumin (ALBU) were found to be significantly altered with a p-value ≤ 0.05 in high grade Meningioma, shown as a heatmap in Fig. S2D. Expression levels of some significant proteins like transferrin, gelsolin and apolipoprotein B have been represented with refined peaks and group-wise box plots. The peptides represented for Transferrin include SASDLTWDNLK and ASYLDCIR, for Gelsolin include TGAQELLR and TASDFITK, and those for Apolipoprotein B TEVIPPLIENR and TSSFALNLPTLPEVK. (Fig. 2D, E, and F). The peak area of all the proteins and the peptides acquired after the analysis is available in Table S5.

### Selection of top features for better segregation of meningioma grades using ML based classification strategy

**Fig. 3:**
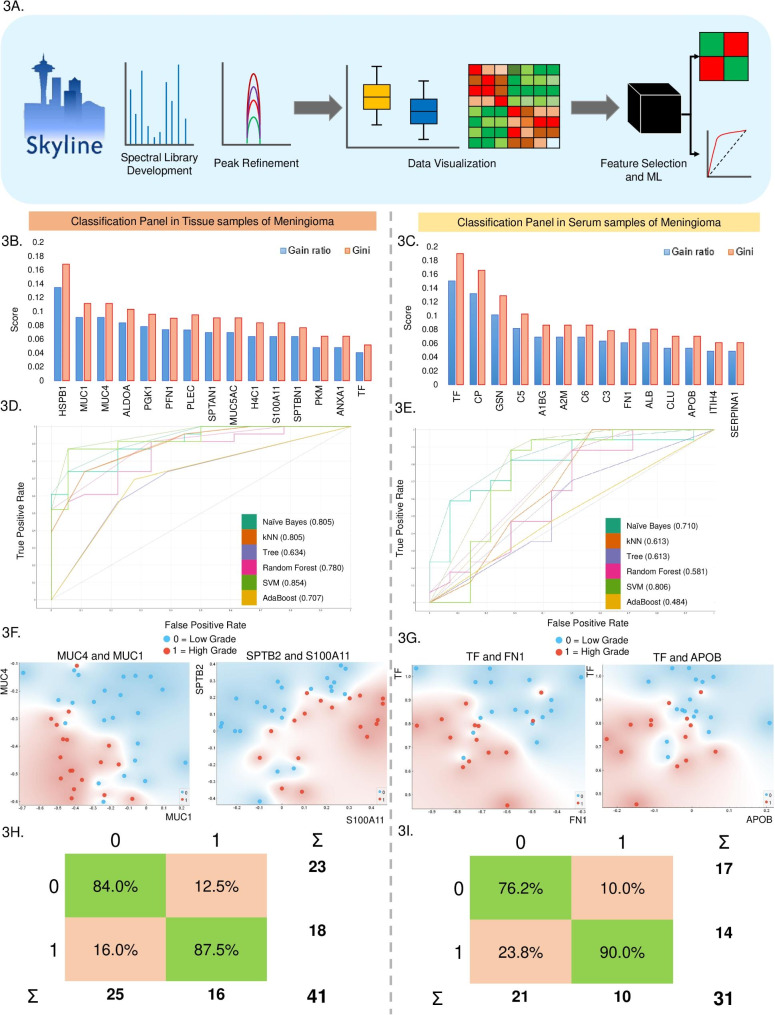
Classification and Segregation potential of selected features using Machine Learning. (**3A**) Schematic outline of Machine Learning and Data analysis workflow. (**3B** and **C**) illustrates the top-ranked features using Gini and Gain ratio in regards to Low Grade and High-Grade segregation in tissue and Serum samples, (**3D** and **E**) Model optimization for serum samples using ROC Curve for Tissue and Serum markers; (**3F**) shows the clustering plot for the combination of MUC4 and MUC1 followed by SPTB2 and S100A11; (**3G**) shows the clustering plot for the combination of TF and FN1 followed by TF and APOB; (**3H** and **I**) represent the confusion matrix using top features for tissue and serum respectively

The list of 49 proteins for tissue and 24 proteins for serum was used for feature selection and ML for which a schematic is provided in Fig. 3A. The analysis has been performed separately for tissue and serum taking only the high grade as group 1 and low grade meningioma samples as group 0. A list of 15 proteins for tissue and 12 proteins for serum were selected based on the Gini index and Gain ratio (Fig. 3B and C). Furthermore, the selected feature was used to build and optimize the classification model to separate the low grade from high grade meningioma. After optimization of the classification models, the SVM showed the best classifier with a mean accuracy of 0.8 in serum and tissue, with a 10-cross-fold validation (Fig. 3D and E). Furthermore, the top feature pair was identified using rank projection and evaluated the clustering of the meningioma samples using Principal Component Analysis (PCA) for both serum and tissue. The analysis showed that a combination of MUC4 and MUC1 followed by SPTB2 and S100A11 showed segregation as a marker pair between low grade and high grade meningioma in tissue. Interestingly, a combination of Transferrin (TF) and FN1 along with TF and APOB showed better segregation as serum markers for meningioma grades. Finally, the performance measurement for ML classification and its accuracy have been shown as confusion plots for both tissue and serum (Fig. 3H and I). A few of these markers, along with the peak area of unique peptides in regard to meningioma grades and spectral library, are shown in Fig. S3A-F.

## Discussion

The increasing incidence of brain tumors and cancer in general has brought the issue of unmet needs in screening, diagnosis and prognostic markers to the forefront. Conventional antibody-based techniques are plagued with issues like low dynamic range, cross-reactivity or availability for timely and efficient management [28] warranting the need to establish alternative laboratory diagnostic modalities. MRM is one such modality with great reproducibility and accuracy [26, 27]. MRM has established its utility in screening, diagnosis, predicting treatment response and prognosis for haematological malignancies and prostate cancer among others. Despite advancements in identifying markers that help improve diagnosis in other cancers, the clinical translation rate for meningioma is very low. There are several studies on meningioma reporting the role of different proteins in meningioma pathophysiology [12, 42, 19, 18, 43, 44]. However, these protein candidates still require validation before deploying them for diagnostic purposes. The current study has been designed with an aim to verify and validate the potential of the previously reported protein markers using targeted proteomics approach for meningioma grade classification. The quantitative data was obtained for 183 unique peptides (corresponding to 49 proteins) for tissue samples, and 118 unique peptides (24 proteins) for the serum samples. Also, among the studied samples, 25 of 41 meningioma tissue and 22 of 32 serum samples were females depicting the disparity in meningioma occurrence across sexes which aligns with the fact that meningiomas are 2½ times more prevalent among females than males [45, 46] .

The peptide level segregation in 53 tissue samples, showed an interesting pattern wherein the control cohort had no overlap with meningioma and glioma cohorts. The glioma cohort was found to have a clear segregation between low grade and high grade meningioma, indicating the potential of these peptides and proteins as meningioma-specific markers. The combination of these peptides show good inter-tumor segregation but requires further validation in a large glioma sample cohort owing to the heterogenous nature of gliomas, especially grade IV gliomas. Notably, between low grade and high grade meningioma, instead of the desired discrete segregation a significant overlap was observed. The overlapping samples in the intersection of the low grade and high grade cohort could help draw multiple inferences. One hypothesis could be that it represents the transition phase between the grades, multiple sub-types, or heterogeneity. However, the list of protein markers has the potential to comprehend and unveil the underlying molecular pattern, thus, assisting in efficient segregation of meningioma tumors. Conversely, the peptide level classification rather than the protein level in 51 serum samples shows better segregation between the low and high grade meningioma with few overlapping samples. Another interesting observation is the overlap and commonalities between normal and low grade meningioma which is also perhaps the reason that early onset and drastic alteration of proteome profile in the bloodstream in the benign stage is not much reported. Similarly, the high grade meningioma cohort had overlap with GBM rather than with meningioma, representing the commonalities between the aggressiveness of the two different and most prevalent brain tumors.

Though the list of all these proteins from both serum and tissues are reported as markers in literature and holds biological relevance, a smaller and more precise panel could help in further validation and clinical implication. Both feature selection and statistical analysis have been performed to select a small subset of 15 and 12 best classifiers for tissue and serum, evaluated using SVM classification strategy. Among the top 15 protein markers identified in tissue samples, in high grade and low grade comparison, Mucins (MUC), Annexin A1 and S100 protein were also present. These are reported IHC markers for meningioma and other cancer diagnoses [47, 48]. Mucin plays an active role in protecting the epithelial cell layers, and different studies suggest their role in malignancy by inhibiting stress-induced apoptosis and regulating gene transcriptions [49]. Clinical reports and studies have validated the importance of Mucin in meningioma diagnosis [50]. Our study identified mucins like MUC1, MUC4, and MUC5A as significantly altered proteins in meningioma grade comparison. In addition, MUC1 and MUC4 were identified as top-ranked classifiers with the best segregation in pair feature projection plots. Annexin A1 (ANXA1) is a known regulator in brain tumors like glioblastoma and assists in tumor immune escape through enhanced IL8 production and NF-kB (p65) activation [51, 26]. We have observed its higher expression levels in high grade samples and high scores of gini index and gain ratio to classify the low and high grades. Another potential cancer marker S100 calcium-binding protein A11 (S100A11), reported in all types of cancer and have a significant correlation with tumor-associated macrophages (TAM), tumor-associated fibroblasts (TAF), and Treg cells [52]. It has also been reported in the context of Meningioma and Glioblastoma tumor progression and unfavorable progression [12] and [53]. In this study, S100A11 was found to be significantly upregulated in high grade meningioma. It also showed maximum segregation between the grades along with Spectrin beta chain (SPTB2) in the pair feature projection plot. Using these diagnostic markers like MUC1, S100A11, and ANXA1, this approach could be tested out as an alternative to confirm the early prognosis of Meningioma.

Apart from these markers, Plectin (PLEC) and Profilin 1 (PFN1) that are previously reported as metastasis regulators in different cancers [54, 55], showed differential expression in our study. Plectin, a cytoskeleton and cell migration marker, have been observed to be down-regulated in our high grade Meningioma samples. In a study by Perez et al., it has been reported that misregulation of plectin induces genomic instability and increased activation could eradicate malignancies [54] and thus referring to a hint that Plectin could be downregulated in high grade tumors. On the other hand, up-regulation of PFN1 in high grade meningioma, could be connected with the stiffness of the extracellular matrix found in aggressive tumor tissues, which promotes proliferation [56]. Moreover, the overall biological network of the proteins such as Aldolase A and Spectrins, featured for ML classification, regulates the Cadherin binding and is involved in cell adhesion.

Serum markers showed better segregation of grades in meningioma and a list of 12 proteins recorded a class accuracy of ~ 80%. We found an upregulation of Transferrin (TF) in low grade patients compared to high grade serum samples. According to the published reports, transferrin receptor 1 showed a higher expression in high grade tumor tissues of meningioma and different cancers [57, 58]. The higher grade of transferrin receptor 1 expression indicates a higher degree of iron consumption and lower levels of transferrin in serum [59]. TF has also shown as one of the promising markers due to better segregation with high accuracy in ML, and could further be correlated with altered iron metabolism and its link with cancer progression and metastasis [60]. Similarly, Apolipoprotein B (APOB), which is significantly altered in meningioma in our study, has been associated with cancers and brain tumors [61, 62]. A study by Zhou et al. illustrated the importance of apolipoprotein profiles and serum ferritin in maintaining homeostasis where the relationship was studied in the context of brain tumors and aggressiveness [63]. Another serum protein found to be significantly altered in our study was Fibronectin (FN1). Though FN1 is altered in malignancies, its role has been poorly understood [64]. The combination of TF and FN1 has been identified as one of the important pair features and showed good classification of meningioma grades. In addition to these markers, we identified Gelsolin (GSN) as a significantly downregulated protein in high grade meningioma. Despite GSN having been reported in meningioma multiple times, there has been no clarity regarding its expression in meningioma grades. However, a study published by Chiu et al. has reported lower expression of circulating GSN in Head and neck cancer and hypothesised that GSN is not only regulating cellular morphology but cell apoptosis as well [65].

In conclusion, we explored and validated the potential of different meningioma biomarkers reported in literature, repositories, and knowledgebases in serum and tissue samples in the Indian population. The clinical diagnosis of meningioma still relies majorly on immunohistochemistry and gene mutation analysis despite the rapid advancement in the field of proteomics. Despite the presence of a long list of proteins with biomarker potential, only a few proteins truly showed potential to segregate the different grades of meningioma. Our observation of transitional tumors exhibiting characteristics of grade I and grade II meningioma in addition to the existing problem of tumor heterogeneity could be a significant obstacle challenging biomarker discovery. However, monitoring serum markers could be a step in the right direction for monitoring the progression of the patients in a non-invasive manner. The successful validation of these proteins in a larger cohort of samples from different populations can help design the panel of protein biomarkers for early diagnosis and prognosis of meningioma.

### Electronic supplementary material

Below is the link to the electronic supplementary material.

Supplementary Material 1

Supplementary Material 2

Supplementary Material 3

Supplementary Material 4

Supplementary Material 5

Supplementary Material 6

## Data Availability

The targeted proteomic datasets are deposited in the PeptideAtlas SRM Experiment Library (PASSEL). 1. Targeted Proteomics Dataset of Serum: http://www.peptideatlas.org/PASS/PASS03806 2. Targeted Proteomics Dataset of Tissue: http://www.peptideatlas.org/PASS/PASS03808
