# Supplementary material for: A large-scale targeted proteomics of serum and tissue shows the utility of classifying high grade and low grade meningioma tumors
Source: Clin Proteomics. 2023 Sep 29;20:41. doi: 10.1186/s12014-023-09426-9 (PMC10540342; doi:10.1186/s12014-023-09426-9)
Supplement: Supplementary file 4 — Supplementary Material 4 [file 12014_2023_9426_MOESM4_ESM.pdf]

Supplementary Figure 1

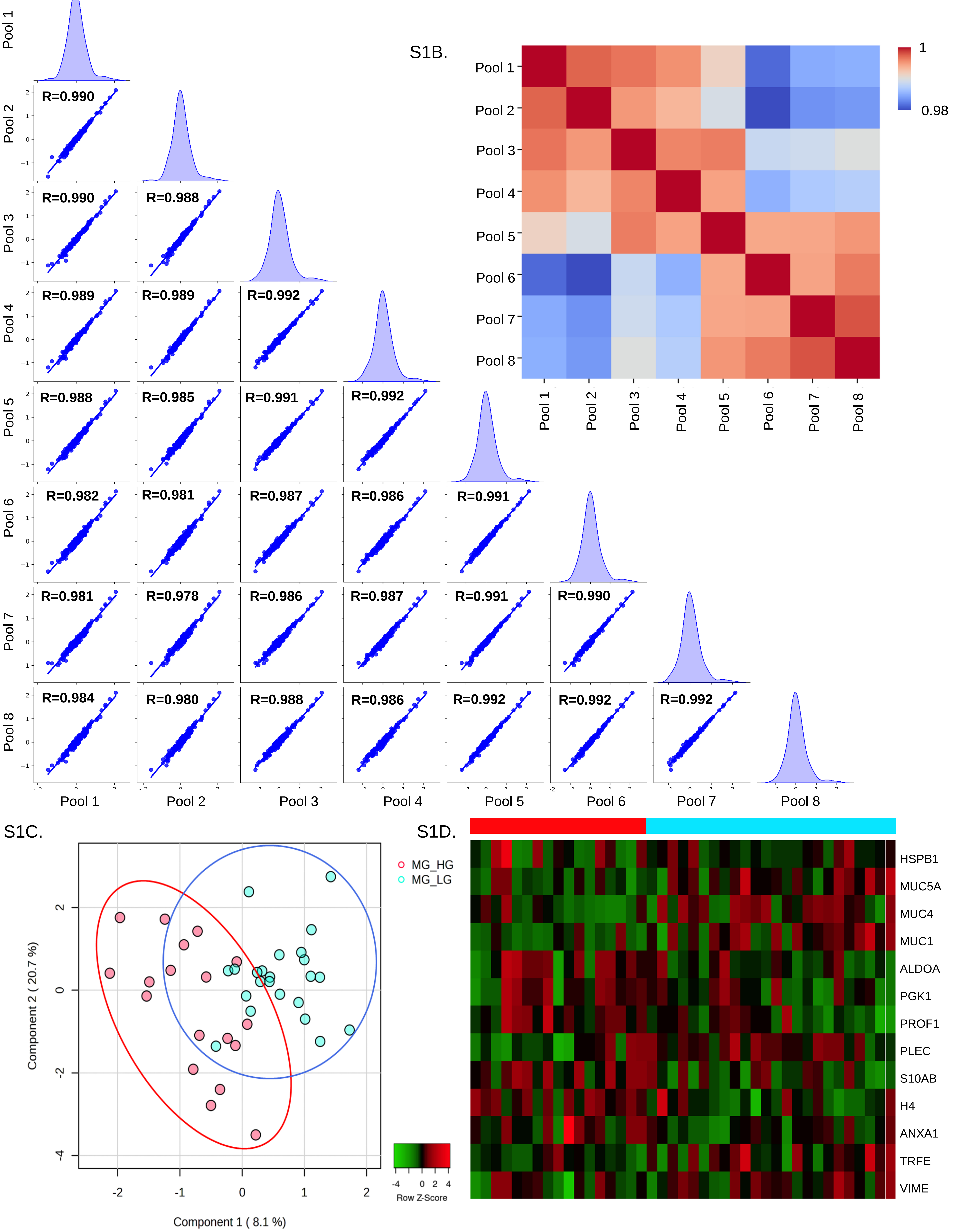

Figure S1 A: Pairplot showing QC Pool correlation of tissue pool samples (peptide) S1B: Heatmap showing QC Pool correlation of tissue pool samples (protein) S1C: PLSDA plot showing segregation between Meningioma High Grade and Low Grade using tumor tissue samples S1D. Heatmap showing the expression protein in different tissue samples of High and Low grade Meningioma

Supplementary Figure 2

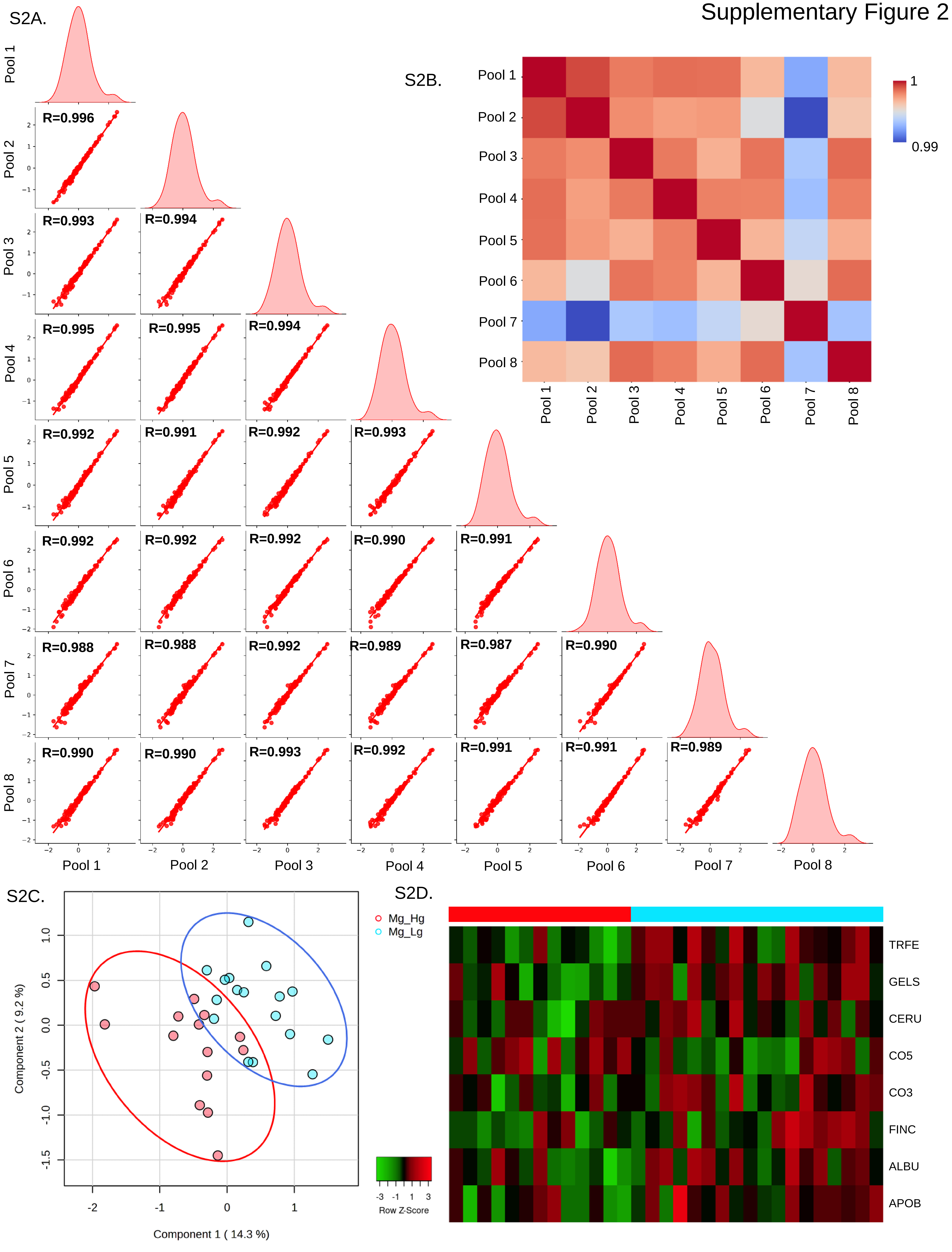

Figure S2 A: Pairplot showing QC Pool correlation of serum pool samples (peptide) S2B: Heatmap showing QC Pool correlation of serum pool samples (protein) S2C: PLSDA plot showing segregation between Meningioma High Grade and Low Grade using serum samples S2D. Heatmap showing the expression protein in different serum samples of High and Low grade Meningioma

Supplementary Figure 3

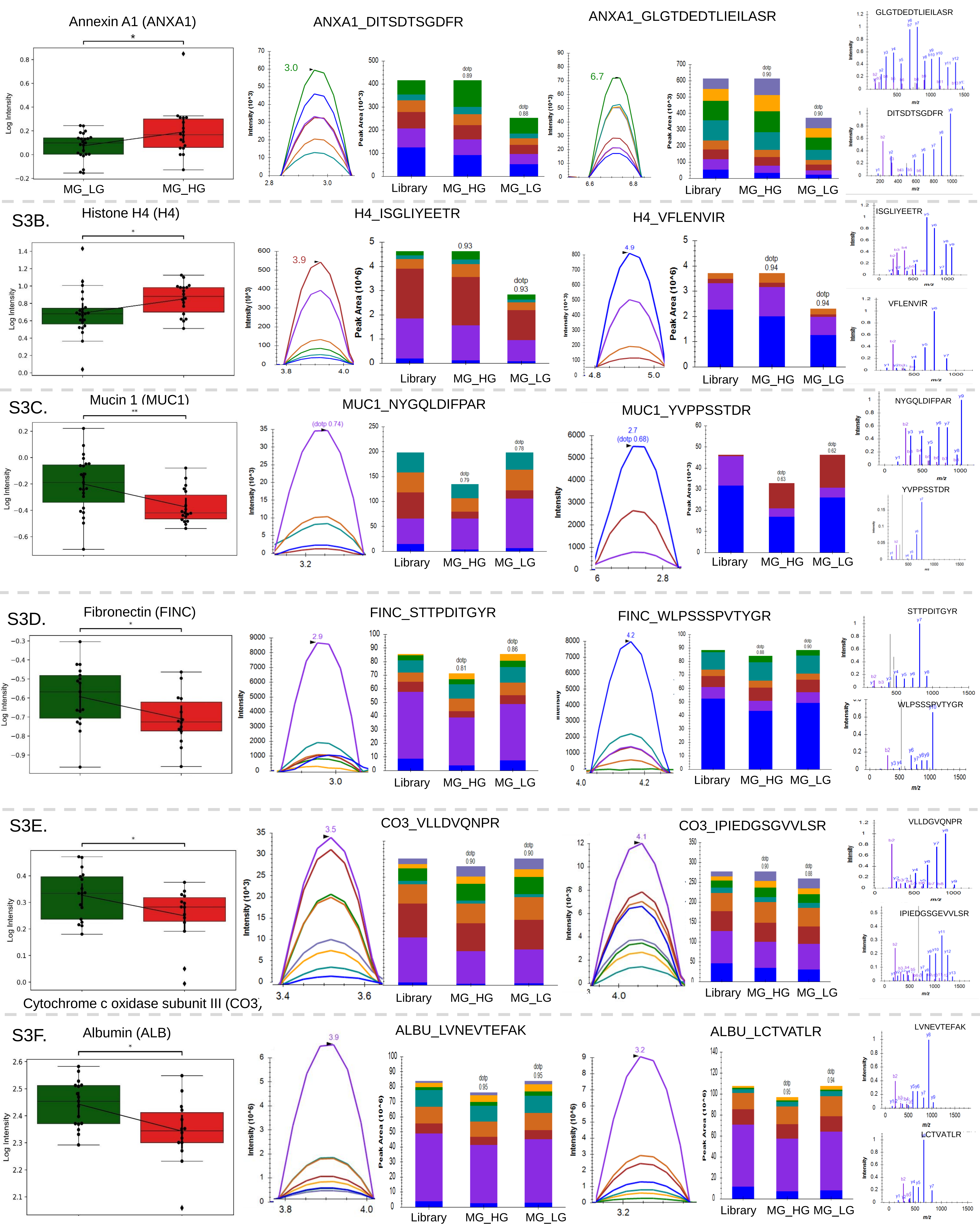

Figure S3A: Box plot showing differential expression of Annexin A1 in high grade and low grade of tumor tissue samples and the bar plots showing peaks along with the peak shape of DITSDTSGDFR and GLGTDEDTLIEILASR peptides matched with their library S3B: Box plot showing differential expression of Histone H4 in high grade and low grade of tumor tissue samples and the bar plots showing peaks along with the peak shape of ISGLIYEETR and VFLENVIR peptides matched with their library S3C:Box plot showing differential expression of Mucin1 MUC1 in high grade and low grade of tumor tissue samples and the bar plots showing peaks along with the peak shape of NYGQLDIFPAR and YVPPSSTDR peptides matched with their library S3D: Box plot showing differential expression of Fibronectin FINC in high grade and low grade of serum samples and the bar plots showing peaks along with the peak shape of STTPDITGYR and WLPSSSPVTYGR peptides matched with their library S3E: Box plot showing differential expression of Cytochrome c oxidase III CO3 in high grade and low grade of serum samples and the bar plots showing peaks along with the peak shape of VLLDVQNPR and IPIEDGSGVVLSR peptides matched with their library.S3F: Box plot showing differential expression of Albumin ALB in high grade and low grade of serum samples and the bar plots showing peaks along with the peak shape of LVNEVTEFAK and LCTVATLR peptides matched with their library

x
